# *Cyphoderus* (Cyphoderidae) as a major component of collembolan cave fauna in Thailand, with description of two new species

**DOI:** 10.3897/zookeys.368.6393

**Published:** 2014-01-07

**Authors:** Sopark Jantarit, Chutamas Satasook, Louis Deharveng

**Affiliations:** 1 Department of Biology, Faculty of Science, Prince of Songkla University, Hat Yai, Songkhla, 90112, Thailand; 2 UMR7205, Origine, Structure et Evolution de la Biodiversité, CP50, Museum National d’Histoire Naturelle, 45 rue Buffon, 75005 Paris, France

**Keywords:** Taxonomy, chaetotaxy, pseudopores, guano

## Abstract

Distinguishing features of *Cyphoderus* Collembola of the *bidenticulati* group are described. Taxonomic problems in the *bidenticulati* group of *Cyphoderus* are emphasized, and new characters of taxonomic value are introduced and discussed. Two new species are described from caves of Thailand, differing mainly in claw morphology.

## Introduction

The species richness of Thai cave faunal communities are poorly known. Most studies in Thailand have focused on low-energy cave habitats, and large regions of the country have seldom been sampled. Consequently, the taxonomy, evolution, and biogeography of Thai cave Collembola are insufficiently known. Surveys of the Thai cave invertebrates revealed that Collembola in the family Cyphoderidae were the dominant arthropods in non-oligotrophic habitats of the dark zone. All examined specimens belong to the *bidenticulati*-group of the genus *Cyphoderus* (*sensu* Delamare-Deboutteville 1948), which previously included 16 species worldwide. Cyphoderidae are typically myrmecophilous or termitophilous, with few records outside of ant and termite nests (Imms 1912, Folsom 1927, Delamare-Deboutteville 1948, Yoshii 1987). The abundance of *Cyphoderus* in caves in the absence or rarity of ants, and the striking morphological similarity of cave forms with myrmecophilous species raises several evolutionary and ecological questions.

Börner (1906) created Cyphoderini as a tribe of Entomobryinae to include *Cyphodeirus albinos* Nicolet, 1842 and three other species that he described in the same paper. In 1913, he upgraded Cyphoderini to subfamily rank, which he placed in Entomobryidae, a concept followed by Delamare-Deboutteville (1948). Subsequently, the taxon was upgraded yet again and was considered a family by most authors (Absolon and Kseneman 1942, Szeptycki 1979, Yoshii 1980, 1987, 1992, Deharveng 2004, Fanciulli et al. 2006). Soto et al. (2008) considered the group to be a subfamily in the Paronellidae on the basis of their non-annulated dens. However, the dens of cyphoderids is clearly reduced in length compared to that of all other Paronellidae *sensu stricto* and always bears characteristic feathered scales (more accurately termed feathered chaetae) consisting of a strong rachis with two symmetrical vanes made of long parallel barbs, a unique structure unknown from other Collembola. On this basis alone, we believe that Cyphoderidae deserve family rather than subfamily status.

Twelve genera have been described in Cyphoderidae (Bellinger et al. 2013): *Calobatinus* Silvestri, 1918 (4 species), *Cephalophilus* Delamare-Deboutteville, 1948 (3 species), *Cyphoda* Delamare-Deboutteville, 1948 (10 species), *Cyphoderinus* Denis, 1942 (1 species), *Cyphoderodes* Silvestri, 1910 (7 species), *Cyphoderus* Nicolet, 1842 (64 species), *Delamareus* Mitra, 1976 (2 species), *Megacyphoderus* Delamare-Deboutteville, 1948 (4 species), *Mimoderus* Yoshii, 1980 (5 species), *Paracyphoderus* Delamare-Deboutteville, 1948 (1 species), *Pseudocyphoderus* Imms, 1912 (4 species) and *Serroderus* Delamare-Deboutteville, 1948 (26 species). The genus *Cyphoderus* is the largest in the family and has a worldwide distribution. Like most cyphoderid species, most *Cyphoderus* species are termitophilous or myrmecophilous (Delamare-Deboutteville 1948, Christiansen 1957, Yoshii 1980, 1987, 1990, 1992). In his extensive revision of Cyphoderidae, Delamare-Deboutteville (1948) divided *Cyphoderus* into 5 groups according to the shape of the mucro (*tridenticulati*, *bidenticulati*, *inermes*, *quadridenticulati* and *multidentati*), to accommodate the 42 species known at that time.

*Cyphoderus* “*bidenticulati*-group” created by Delamare-Deboutteville (1948) and studied in this paper are easily recognized by their long, thin, and yellow mucro ending in two subequal small teeth. This group includes a large number of forms described as species, several only known from a single location, and a few species given as widespread on the account of numerous literature records. However, most of these records are doubtful because most species in this complex lack conspicuous morphological features, and are therefore difficult to distinguish.

Not only the so-called species are difficult to separate, but the description of the taxon’s widespread type species, *Cyphoderus albinus* Nicolet, 1842, is poor by modern standards. In fact, the original description of Nicolet (1842) is so vague that it could apply to almost any species in the *bidenticulati* group. The most reliable, recent information comes from three sources: Delamare-Deboutteville (1948), whose description is probably based on French material; Yoshii (1990) based on material of Macaronesia; and Fjellberg (2007) describing material from Scandinavia. However, these contradictory accounts add further confusion, as there are disagreements about major diagnostic characters. According to Delamare-Deboutteville (1948), the species has no unpaired inner tooth on claw; the other two descriptions mention one unpaired tooth, but not at the same level. Fjellberg (2007) stated that there is no sublobal hair on outer maxillary lobe; Yoshii figured one. These contradictions may represent variability among populations, different species placed under the same name, or inaccurate observations. The only certainty is that the *bidenticulati* group of *Cyphoderus* is a complex of extremely similar forms after Delamare-Deboutteville (1948), where morphological examination reaches its limit for delimiting species. In this paper we describe new morphological characters, beyond those introduced by Yoshii, and provide detailed descriptions that could serve as references for future taxonomic works. The redescription of type material or topotypes will be necessary to extend the present work. In parallel, the use of molecular taxonomy might be the easiest way to assess the status of populations.

## Materials and methods

**Figure 1.**
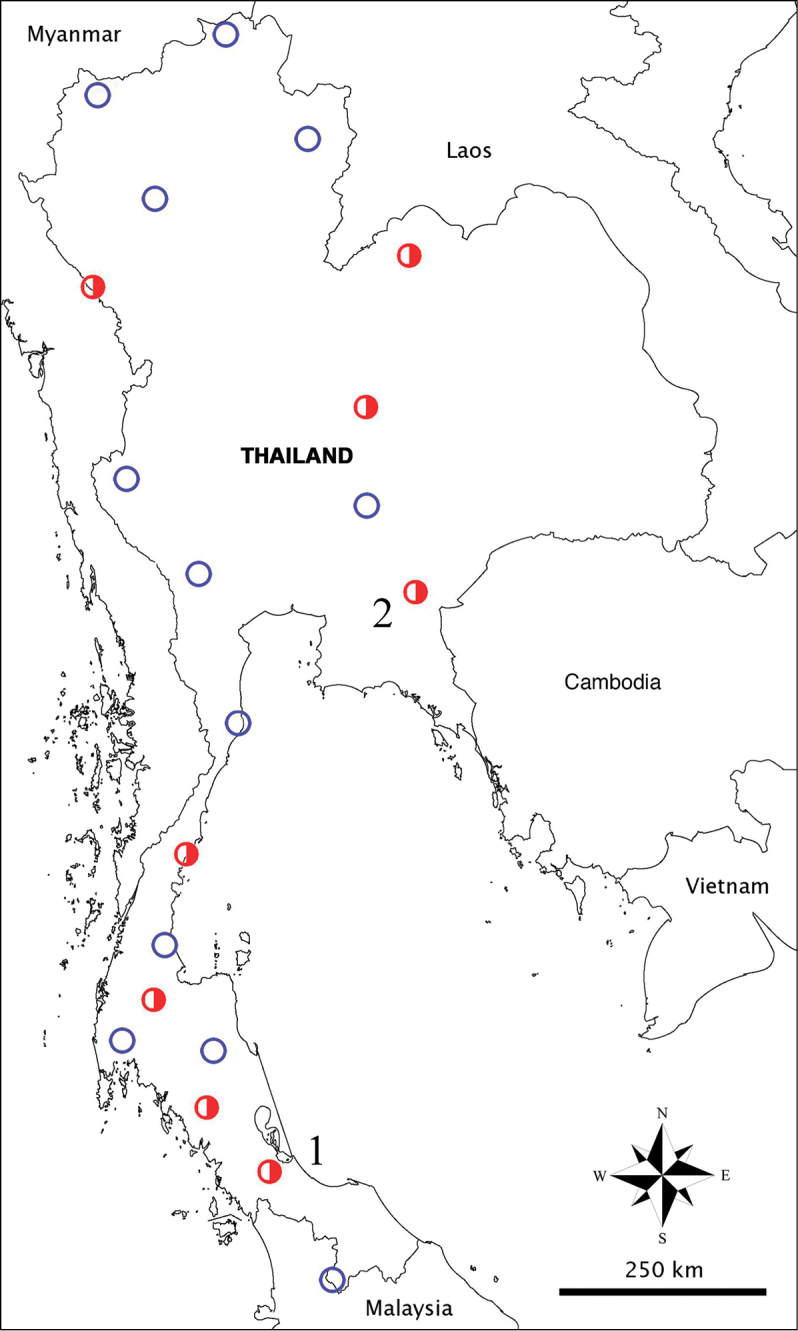
Sampling locations of cave Cyphoderidae in Thailand. Blue empty circles = caves without cyphoderids; red half–circles = caves with cyphoderids; C1, *Cyphoderus songkhlaensis* sp. n.; C2, unidentified species (Tham Nam Pray, Huay Yod District, Trang Province); C3, unidentified species (Tham Phung, Kiri Rat Nikhom District, Surat Thani Province); C4, unidentified species (Tham Phra, Patil District, Chumphon Province); C5, *Cyphoderus khaochakanus* sp. n., C6, unidentified species (Tham Kaeo, Pakdee Chumphon District, Chaiyaphum Province); C7, unidentified species (Tham Phupha Yatha Wararam, Muang Loei District, Loei Province); C8, unidentified species (Tham Mae U-Su, Tha Song Yang District, Tak Province).

Collembola were extracted from cave substrate samples using Berlese funnels and pitfall traps and stored in 90% ethanol at 5°C. Caves were sampled throughout Thailand (Fig. 1). The two described species come from two caves that yielded abundant populations, one from eastern Thailand and the other from the peninsula. Specimens were cleared in lactic acid and mounted on slides in Marc Andre II gum. The morphological analyses used a Leica DMLB light microscope. Images taken on a Cambridge 600 scanning electron microscope (SEM) were used for intepretating fine morphology of some chaetae. Figures were improved with Photoshop CS5 (Adobe Inc.).

### Material deposition

PSU Prince of Songkla University, Hat Yai, Songkhla, Thailand

MNHN Muséum national d’Histoire naturelle, Paris, France

Abbreviations used in the descriptions: Abd. = abdominal segment; Th. = thoracic segment; Ant. = antennal segment; AIIIO = Ant.III organ; M (in figures) = macrochaeta(e); mac (in text) = macrochaeta(e); mes = mesochaeta(e); mic = microchaeta(e); sens = S-chaeta; T (in figures) = trichobothria; Man = manubrium (in tables). Chaetae notation: frontal chaetae of head and ventral tube chaetae after Yoshii (1980), tergite chaetotaxy after Szeptycki (1979), labial palp after Fjellberg (1999), AIIIO and ventral cephalic chaetae after Chen and Christiansen (1993).

## Systematics

### Cyphoderus

Nicolet, 1842

http://species-id.net/wiki/Cyphoderus

#### Type species.

*Cyphoderus albinus* Nicolet, 1842

#### Character assessment.

Several characters of taxonomic importance were discovered or re-appraised in the course of this study.

**Figure 2.**
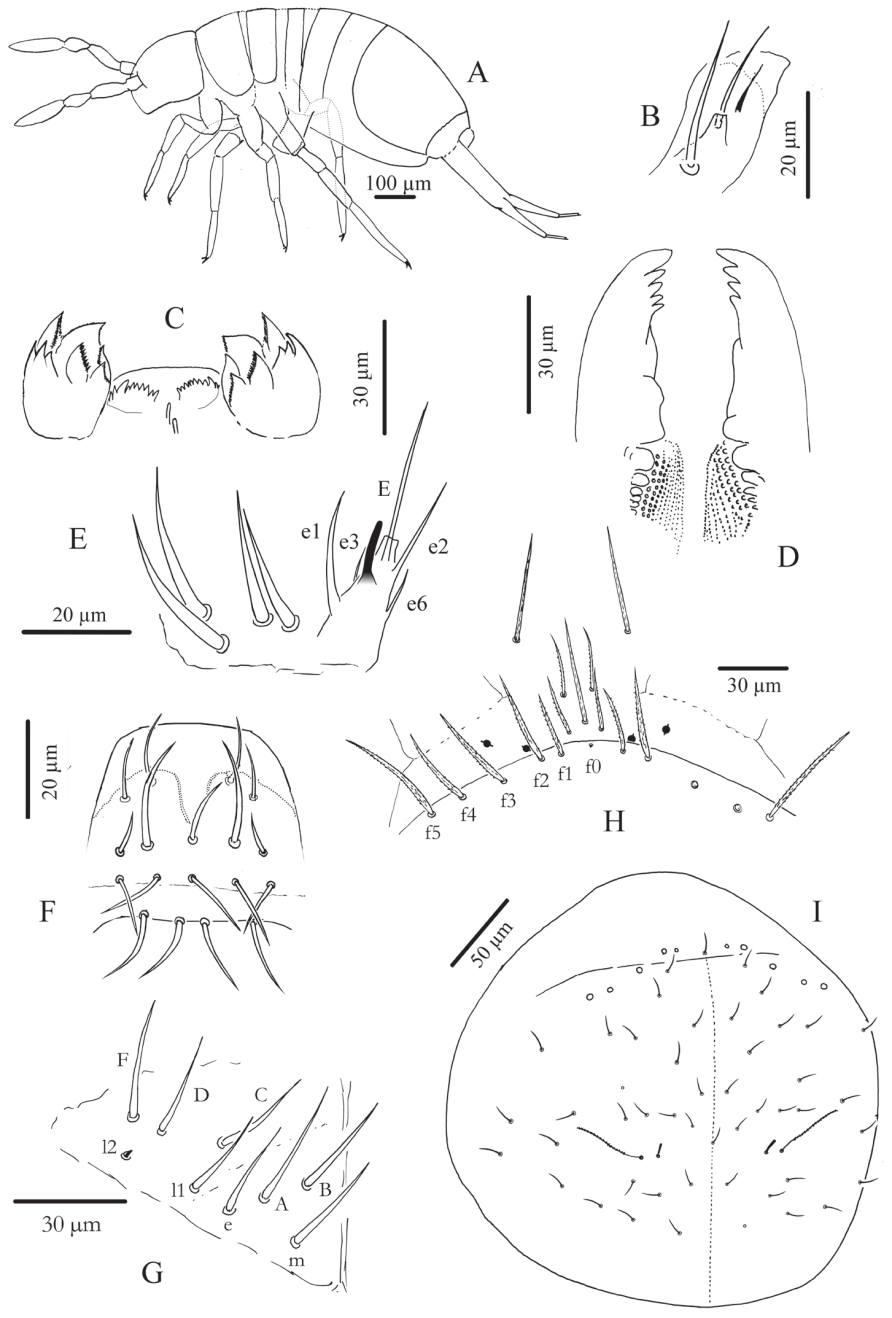
*Cyphoderus songkhlaensis* sp. n. **A** habitus **B** outer maxillary lobe **C** maxilla head and ventral complex of the labrum **D** mandible **E** labial palp: proximal chaetae and external papilla E **F** labrum, dorsal view **G** chaetotaxy of labial basis; frontal chaetae **H** frontal chaetae and pseudopores of head **I** dorsal chaetotaxy of head.

**Figure 3.**
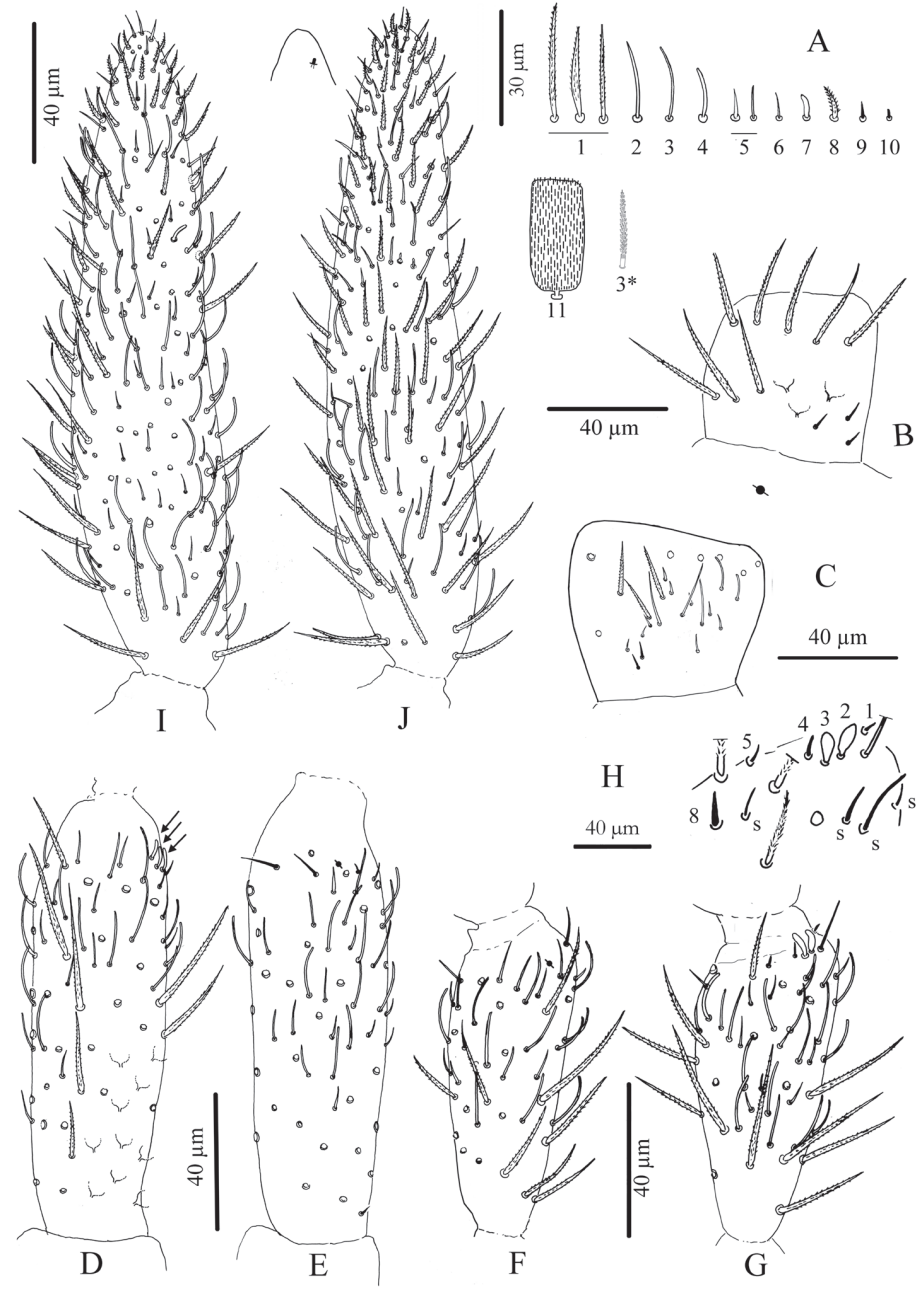
*Cyphoderus songkhlaensis* sp. n. continued **A** chaetae of antenna drawn from optical microscope, except 3* derived from SEM image **B** dorsal side of right Ant.I **C** ventral side of right Ant.I **D** dorsal side of right Ant.II; the apical swollen sens of type-7 are indicated by arrows **E** ventral side of right Ant.II with apical pseudopore **F** ventral side of right Ant.III with apical pseudopore **G** dorsal side of right Ant.III **H** distal organite of Ant.III **I** ventral side of Ant.IV **J** dorsal side of Ant.IV with separate view of the subapical organite (left).

**Figure 4.**
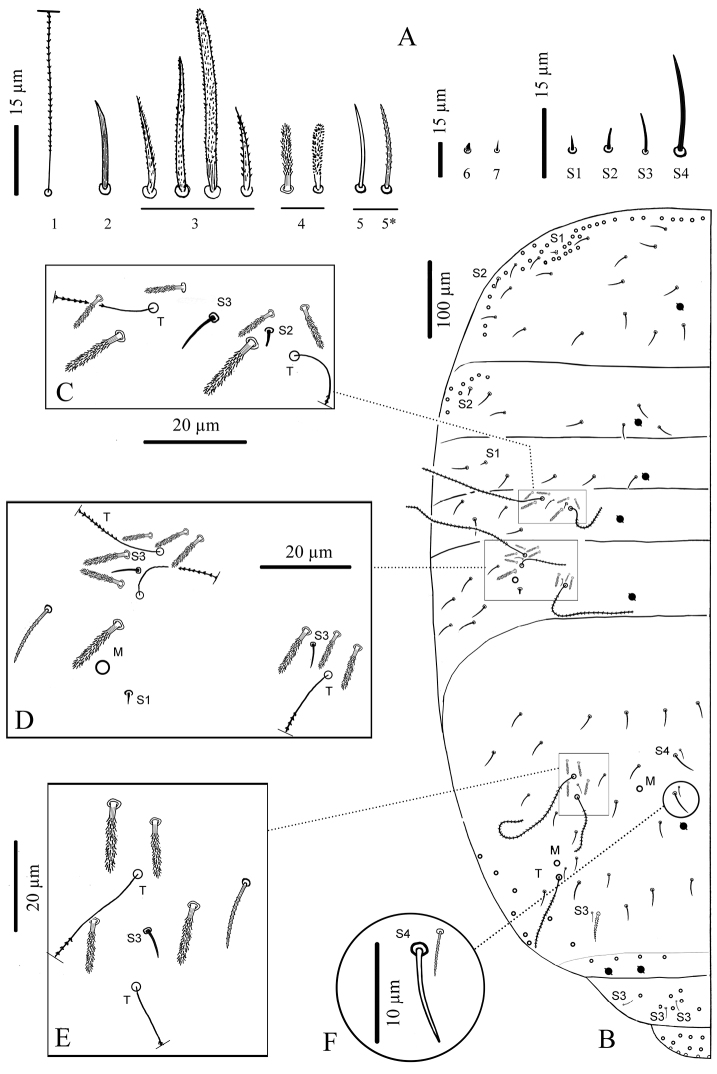
*Cyphoderus songkhlaensis* sp. n. continued **A** chaetae of tergites drawn from optical microscope, except 5* derived from SEM image **B** chaetotaxy of tergites with types of S-chaetae S1 to S4 **C** trichobothrial complexes of Abd.II **D** trichobothrial complexes of Abd.III **E** anterior trichobothrial complexes of Abd.IV **F** tandem of chaetae on Abd.IV; the smallest is a short type-5 mes and the largest a S4 sens.

**Figure 5.**
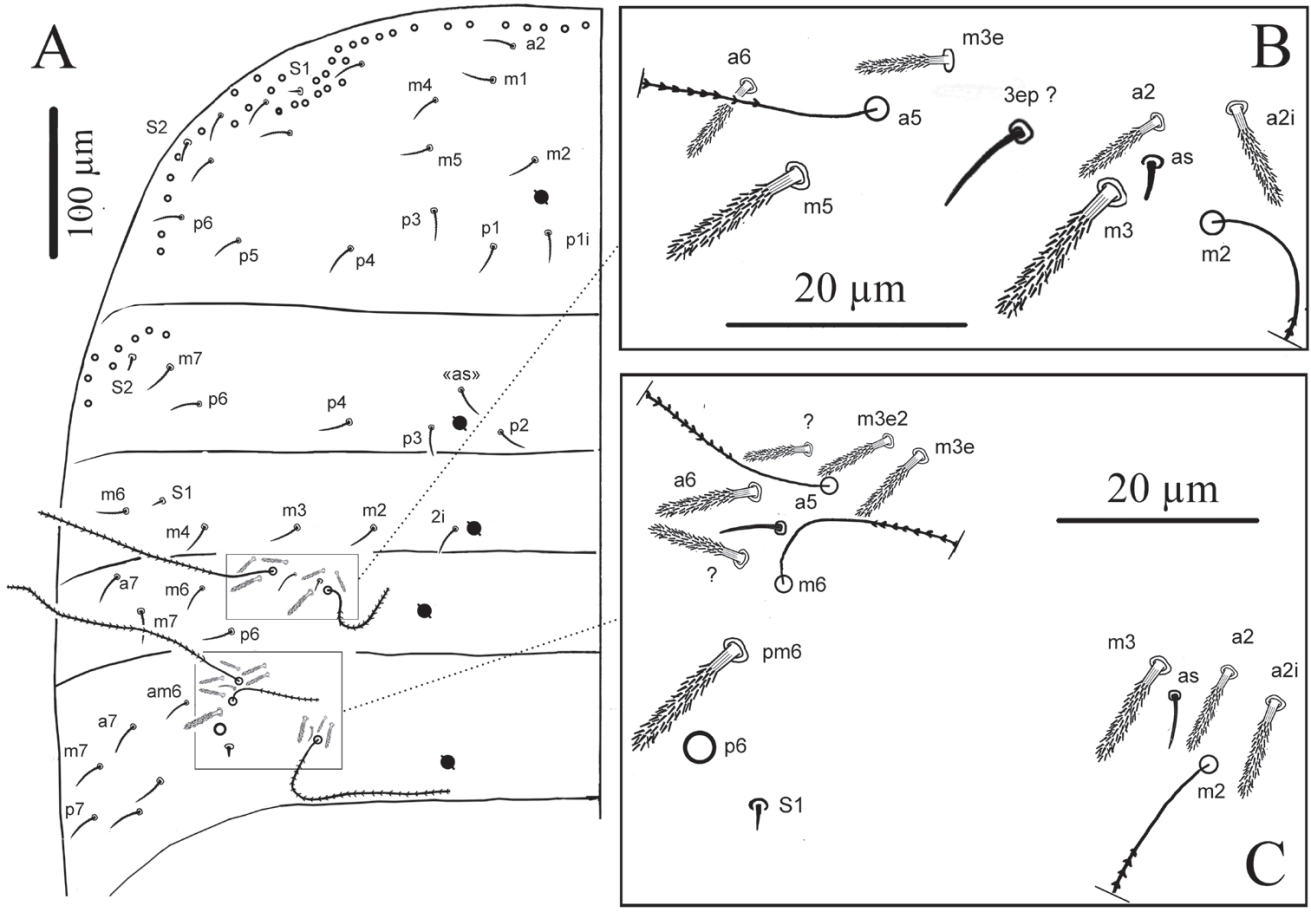
*Cyphoderus songkhlaensis* sp. n. continued **A** Szeptycki’s notation of tergal chaetae on Th.II-Abd.III (Szeptycki 1979) **B** detail of Abd.II trichobothrial area **C** detail of Abd.III trichobothrial area.

**Figure 6.**
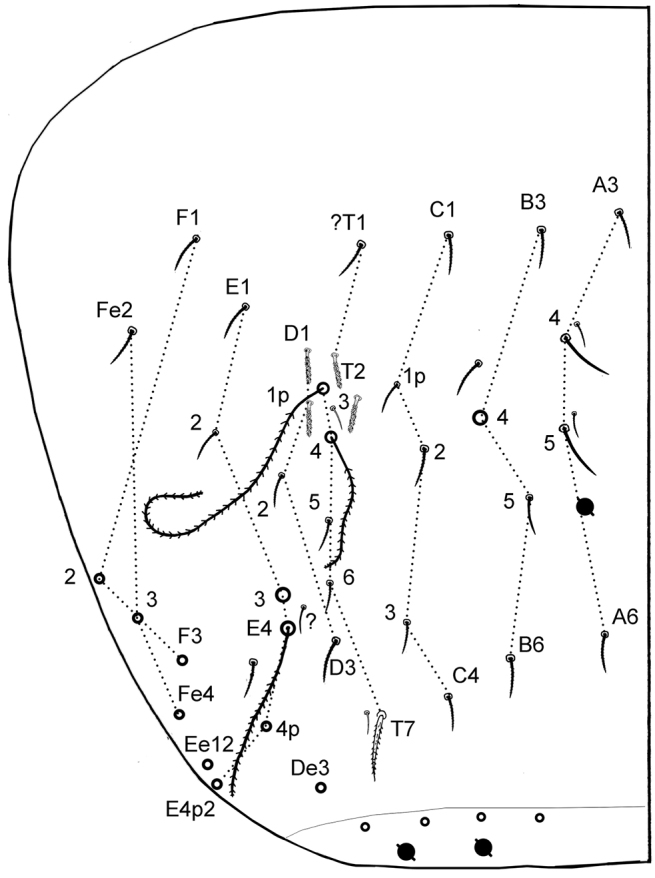
*Cyphoderus songkhlaensis* sp. n. continued, Szeptycki’s notation of tergal chaetae on Abd.IV (Szeptycki 1979).

**Figure 7.**
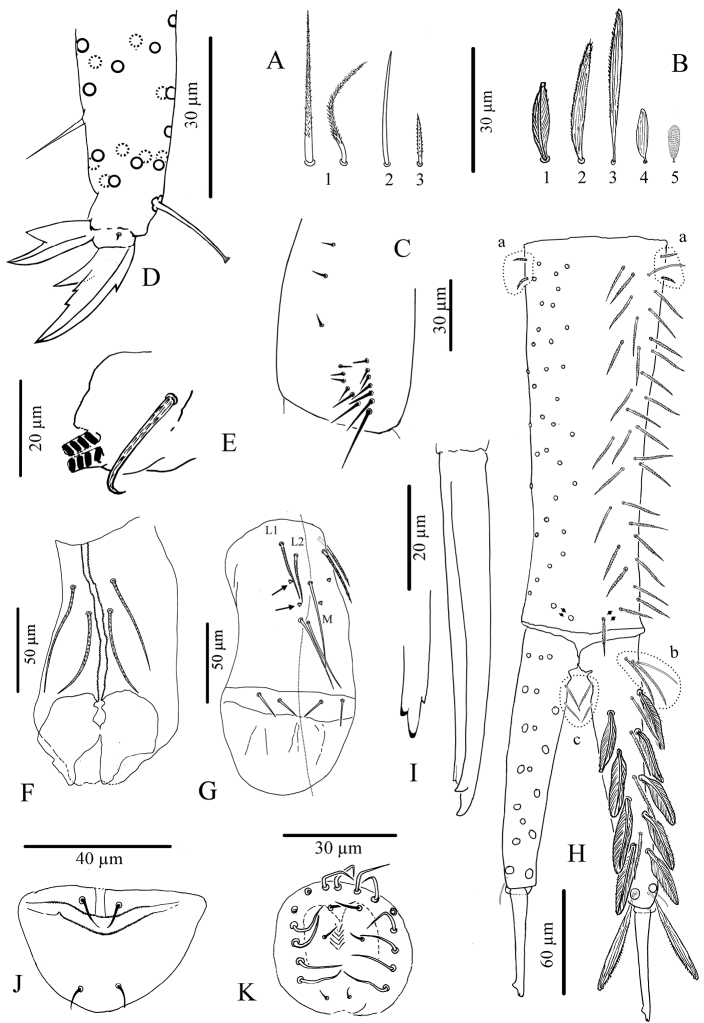
*Cyphoderus songkhlaensis* sp. n. continued **A** chaetae of furca **B** scales of furca **C** trochanteral organ **D** claw and distal part of tibiotarsus III **E** tenaculum **F** anterior face of the ventral tube **G** posterior face of the ventral tube; the peg-like setulae are indicated by arrows **H** furca; encircled by dotted lines are the 2+2 latero-basal mesochaetae of manubrium (**a**) the 3 outer basal mesochaetae of dens (**b**) and the 2+2 inner basal mesochaetae of dens (**c**) (I) mucro in lateral view (right) and in dorsal view (left) showing a third minute external tooth **J** female genital plate **K** male genital plate.

1) All antennal segments were examined on both dorsal and ventral sides, revealing 10 types of chaetae (Fig. 3A). Their distribution pattern on the antennae is complex, but similar in the two species. Similarities are also obvious with the few Entomobryoidea where antennal chaetotaxy has been described. For instance, sens 1 to 5 and 8 of AIIIO as figured in *Sinella* by Chen and Christiansen (1993) were easily retrieved in our *Cyphoderus* (Fig. 3G). Several of the chaetal types recognized here are also found in other genera of Entomobryoidea. However, patterns are very complex and their comparisons would require detailed analyses beyond the scope of this paper.
2) S-chaetae can be grouped in four types (Fig. 4A), with chaeta S4 difficult to distinguish from type-5 mes. The S-chaetae formula observed in our species, as well as in other unidentified ones of the *bidenticulati* group, is 0/2,1/1,2,3,4,3,0 from head to Abd.VI (Figs 4–6), including 0/1,0/1,0,1,0,0 for S1; 0/1,1/0,1,0,0,0 for S2, 0/0,0/0,1,2,2,3 for S3 and 0/0,0/0,0,0,2,0 for S4. This S-chaetae pattern is similar to that of Entomobryoidea, except for the position of chaetae S1 and S2 on Th.II. In Entomobryoidea, S1 and S2 (=ms and S in Zhang et al. 2009) are close each other antero-laterally on the tergite (see Zhang et al. 2009). In the examined *Cyphoderus*, S2 is not close to S1, but intermediate between the position of antero-lateral S2 and of the postero-lateral S2 as observed in several Entomobryidae.
3) Pseudopores on tergites are arranged as in the Entomobryoidea species where they have been recorded (Jordana 2012 for instance): 1,1/1,1,1,1,0,0 from Th.II to Abd.VI. The presence of dorso-distal pseudopores on manubrium (2+2 in the studied *Cyphoderus*, Fig. 7H, 8D) is also characteristic of Entomobryoidea. Special to *Cyphoderus* described here are the 2+2 pseudopores behind the posterior row of chaetae of Abd.IV, found also in other unidentified *Cyphoderus* of the *bidenticulati* group (Fig. 4B). This pseudopore location is only known in Troglopedetinae, *i.e*., in *Troglopedetes* (Deharveng 1988), in *Cyphoderopsis* (Jantarit et al. 2013) and in *Trogolaphysa* (Soto-Adames and Taylor 2013), with a number of pseudopores different for each genus. A ventral pseudopore is present on antennal area, in the same location as in Isotomidae (Deharveng 1979), Neanuridae (Deharveng 1983) and Onychiuridae (Pomorski 1998, Martinez et al. 2004). At least, the presence of 1+1 or 2+2 pseudopores on head anteriorly to the antenno-basal line (Fig. 2H) is a new pseudopore location for Collembola, unnoticed as far as we know in other genera of the class.
4) Important features of dorsal head chaetotaxy have been discovered by Yoshii (1980, 1987, 1992), useful for characterizing the family Cyphoderidae and several taxa of lower rank. The number and arrangement of post-labial chaetae as well as the presence of one mic among them are the same in the two species described here. However, they differ when compared with other species and might provide another promising set of taxonomic characters.
5) Body chaetae of various types were detected and tentatively grouped in categories. The mes of type-5 are the most numerous chaetae dorsally. They are seen as smooth under microscope examination, but serrated under SEM, Fig. 4A5; distinguishing them from S4 sens is especially difficult on Abd.IV where both are present, and the same confusion may arise for many other Entomobryoidea. As patterns of these mes as well as those of S4 sens seem to be stable inside population and different between species, further investigations will have to re-examine this character for its use in taxonomy.
6) The chaetotaxy of dorsal side (Figs 4–6) matches in most cases that given by Szeptycki (1979) for *Cyphoderus albinus*, and is very similar to that of Entomobryoidea (see Zhang et al. 2009). Main differences include the relative position of S1 and S2 on Th.II (see above), and chaeta “as” of Th.III as a mes in our material versus a short S-chaetae in Szeptycki (1979).
7) One of the most important characters for differentiating species of the *bidenticulati* group is claw morphology, and it is the most diagnostic feature of the species described here. Although some variability in size and position of the various dental teeth has been noticed by other authors, it has not been taken into account in previous descriptions, leaving doubts about the validity of several species.

### Cyphoderus songkhlaensis sp. n.

http://zoobank.org/99107FAB-981B-4F23-9D87-B962FEA5DB7A

http://species-id.net/wiki/Cyphoderus_songkhlaensis

Figs 2 –7

#### Type locality.

Thailand: Songkhla Province, Rattaphum District, Tham Khao Nui (12°12.227'N, 99°59.524'E), 120 m. above sea level, dark zone in cave, pitfall traps and Berlese extraction, S. Jantarit leg, 05 May 2012 (Sample #Songkhla-SJ.001).

#### Type material.

Holotype, male adult (#PSUZC2011.SONG-001H) and 44 paratypes (6 males, 3 females, 35 of unknown gender) mounted on slides. Holotype and 29 paratypes at PSU (25 slides, 4 males, 2 females and 23 subadults, collection #PSUZC2011.SONG-001P-030P) and 15 paratypes at MNHN (12 slides, 2 males, 1 female and 12 subadults).

#### Description.

Habitus thick (Fig. 2A), not troglomorphic, body length about 1.2 mm excluding antennae and furca. Furca well developed, about 2.5 times shorter than body. Body color white. Eyes absent, no ocular patch. Dense cover of scales on head, body and furca (ventrally on manubrium, both sides on dens); scales present on Ant.I–II dorsally, absent on legs and ventral tube. Four categories of chaetae: ordinary chaetae (mac, mes and mic), scales, trichobothria and S-chaetae (= sens), described below separately for antennae and body.

**Mouthparts.** Outer maxillary lobe with one basal chaeta, a simple palp and one sublobal hair (Fig. 2B). Maxilla with 3-toothed capitulum and complex of 5 pad-shaped lamellae not analyzed in detail (Fig. 2C). Mandible head stocky, asymmetrical with 5 (left) and 4 (right) teeth (Fig. 2D). Labial palp with 5 papillae (A-E) and 13 guards, exactly as figured by Fjellberg (1999: fig. 72) (A and C without guards, B with 5 guards, D and E with 4 guards each); three hypostomal chaetae present with H longer than h1 and h2; 4 proximal chaetae (Fig. 2E). Labial basis formula m, e, l1, l2, with all chaetae smooth or indistinctly serrated, and l2 reduced to a minute but thick mic (Fig. 2G). Labral formula 4/5,5,4 with all chaetae smooth; two chaetae of the mid-row stronger and longer than others; dorso-distal limit of primary granules with a deep central incision (Fig. 2F); labral edge without structure; ventro-distally, two asymmetrical combs with many teeth variously developed and two central tubules (Fig. 2C).

**Antennae.** Less than 2 times the length of the head, segmentations I: II: III: IV as 1: 2.7: 1.6: 3.9. Sens and sens-like chaetae present on all antennal segments, of 10 morphological types (Fig. 3A); type-3 mes rather long, smooth under microscope examination but ciliated under SEM as in Fig. 3A (type-3*). Scales present dorsally on Ant.I and II (Fig. 3A11). Ant.I dorsally (Fig. 3B) with scales and ciliated mes (type-1), except 3 basal mic (type-9); ventrally (Fig. 3C) with various types of chaetae (types-1,2,3,5,6 and 9). Ant.II (Fig. 3D, E) dorsally and ventrally with numerous slender sens and chaetae (types-1,3,4,5,6,7); scales present dorso-basally; distally, 3–4 dorso-external swollen sens of type-7 and one ventro-external pseudopore. Ant.III (Fig. 3F, G) with various types of chaetae (types-1,3,4,5,6,7,9,10) not analyzed in detail; dorso-externally, AIIIO (Fig. 3H) typical of Entomobryoidea, with sens 1 to 5 and 8easily recognized, 2 and 3 being swollen sens of type-7; ventro-externally, one subapical pseudopore. Ant.IV (Fig. 3I, J) devoid of apical bulb, with various types of chaetae (including all types of sens except type-10); subapical organ present dorso-externally as a short and thick rod.

**Body chaetae** (Fig. 4A).

1) trichobothria, ciliated, very long and thin
2) weakly serrated, spiny mes
3) serrated or ciliated chaetae, of various length (mes to mac) and thickness
4) short and thickened mes in trichobothrial areas
5) thin mes, smooth under microscope examination, but ciliated under SEM
6) thick minute mic f0 and X on head
7) thin minute mic of anal valves
S1) smooth, dark, short, straight, pointed sens
S2) smooth, hyaline, short, subcylindrical, blunt sens
S3) smooth, hyaline, longer, thinner sens
S4) smooth, rather long, rather thick, blunt sens

Scales oval to rectangular in shape, of various size, covering the whole body dorsally.

**Dorsal chaetotaxy and pseudopore patterns (per side).**

Macrochaetae: 0/0,0/0,0,1,2 from head to Abd.IV (excluding the antenno-basal lines on head and the 7–8+7–8 lateral mac on Abd.IV) (Fig. 4B).

Type-5 mes: 24–25 (and 1 uneven) /16,6/5,4,6,22,0,0 from head to Abd.VI (approximate numbers) (Fig. 2I for the head).

Trichobothria: 1/0,0/0,2,3,3,0,0 from head to Abd.VI.

S-chaetae (sens of types S1, S2, S3 and S4): 0/2,1/1,2,3,4,3,0 from head to Abd.VI. Possibly more on Abd.IV where type-5-like mes are often difficult to separate from S4.

Pseudopores: 1–2/1,1/1,1,1,1+2,0,0 from head to Abd.VI.

**Chaetotaxy and pseudopores on head.** As in Fig. 2I (dorsal side). No dorsal mac except the antenno-basal line of 5 mac (f1-f5); f0 as a minute thick uneven mic of type-6 between f1 chaetae; five ciliated clypeal mes and 1–2+1–2 pseudopores anteriorly to f1 (Fig. 2H). About 24 dorsal cephalic mes of type-5, subequal, short (Fig. 4A5). Cephalic trichobothria present dorsally at the middle of head with 1+1 mes internally near trichobothria, short and feebly ciliated (Fig. 2I, similar to Fig. 4A3). Ventrally, 4+4 post-labial mes smooth or very finely serrated along linea ventralis, and one mic of type-6 between G3 and H3 probably homologous with X (Fig. 2 in Chen and Christiansen 1993).

**Chaetotaxy and pseudopores per tergite.** (Figs 4B–F); values for type-5 mes are indicative). Th.II without mac; with a row of subequal spiny mes anteriorly and laterally, and several rows antero-laterally (type-2), 1+1 antero-lateral sens S1, 1+1 lateral sens S2 not close to S1, about 16+16 mes of type-5, and 1+1 pseudopores close to axis. Th.III without mac; with 1+1 antero-lateral sens S2, about 6+6 mes of type-5, and 1+1 pseudopores.

Abd.I without mac; with 1+1 lateral sens S1, about 5+5 mes of type-5 and 1+1 pseudopores.

Abd.II without mac; with 2+2 trichobothria, 6+6 modified mes around the trichobothria (type-4, Fig. 4C), 1+1 sens S2 (Fig. 4C) and 1+1sens S3 (Fig. 4C), about 4+4 mes of type-5, and 1+1 pseudopores. Abd.III with 3+3 trichobothria, 1+1 mac, 9+9 modified mes of type-4 on trichobothrial areas (3+3 near the internal trichobothria and 6+6 near the two external trichobothria, Fig. 4D), 3+3 sens in trichobothrial areas (1+1 S1 and 2+2 S3, Fig. 4D), about 6+6 mes of type-5, and 1+1 pseudopores. Abd.IV with 3+3 trichobothria, 2+2 mac, 4+4 modified mes of type-4 in the anterior trichobothrial area (none in the posterior trichobothrial area, Figs 4E and 6), 2+2 sens S3, 2+2 sens S4 near axis, about 22–23+22–23 mes, 2+2 sens S4 ahead pseudopores, in tandem with 2+2 short probably type-5 mes (Fig. 4F), 1+1 serrated mes of type-3 in tandem with 1+1 sens S3 posteriorly, and 3+3 pseudopores (1+1 in the middle of Abd.IV, 2+2 in the posterior margin of the tergite, behind a posterior row of 4+4 mes). Abd.V without pseudopore or mes of type-5; with 3+3 sens S3 and several short mac and mes.

**Legs.** Without scales. Trochanteral organ with 11–22 simple, straight, smooth chaetae arranged in V-form (Fig. 7C). Tibiotarsus chaetotaxy mostly composed of strong ciliated mes, with one thick smooth ventro-subapical chaeta on hind tibiotarsus. Each tibiotarsus with one tenent hair rather stout, apically spatulated, 4/5 as long as inner edge of claw; distal row of 9–10 serrated chaetae irregularly arranged on all tibiotarsi (Fig. 7D). Claw broad, not slender, with a weak or inconspicuous tunica; with one tooth at 40% of inner edge from the tip of the claw, a small dorsal tooth basally and a pair of inner basal teeth of unequal size, the outer one much larger than the inner one (Fig. 7D). Unguiculus pointed and broad, more than half as long as claw, lanceolate, with a strong outer tooth (Fig. 7D).

**Ventral tube.** Without scales. Anterior face with 2+2 long serrated chaetae (Fig. 7F). Posterior chaetae arranged typically for the genus, with L1 and L2 ciliated, L2 shorter than L1, M elongate and smooth, accompanied by 2+2 small peg-like microchaetae, and two long smooth distal chaetae; lateral flaps each with 2 small smooth mes (Fig. 7G).

**Furca.** Tenaculum with 4 teeth on each branch, anteriorly with strong, densely serrated, bent uneven chaeta (Fig. 7E). Furca with three types of chaetae (Fig. 7A) and 5 types of scales (Fig. 7B). Manubrium about 1.2 times as long as mucrodens. Dens about 2.3 times as long as mucro. Dorsal side of manubrium (Fig. 7H) with 2+2 pseudopores distally, and about 32–35 mes (fallen in most cases) arranged in two longitudinal stripes, including rather flexible and strongly ciliated mes and a few lateral ones slightly stronger, more straight, feebly serrated (Fig. 7A type-1), and baso-laterally 2+2 short serrated mes (Fig. 7A type-3); ventral side covered with oval scales (Fig. 7B type-5). Dens (Fig. 7H) elongate, dorsally with 2 rows of feathered scales (Fig. 7B type-1), 6 external and 5 internal, and 4 ciliated mes (Fig. 7A type-1) between two rows; proximal outer part of dens with 3 chaetae, two ciliated (Fig. 7A type-1) and the most external one smooth (Fig. 7A type-2); proximal inner part of dens with 2 slightly serrated mes (Fig. 7A1) close to dens-manubrium articulation; apical outer part of dens with one short serrated mes (Fig. 7A type-3); long dorso-distal feathered scales fallen in our specimens. Dens ventrally with oval scales (Fig. 7B types-4, 5), the distal internal one almost as long as mucro (Fig. 7B2, 3). Mucro straight, elongate, bidentate apically, with one minute external tooth almost at the level of the ante-apical normal tooth (Fig. 7I).

**Genital plate.** Male genital plate of the circinate type (*sensu* Christiansen 1958), with 6 genital mic and 15–16 circumgenital short, thin, smooth mes (Fig. 7K). Female genital plate with 2+2 mic (Fig. 7J).

#### Measurement.

in µm (from type specimen #PSUZC2011.SONG-001H, male).

**Table:** 

**Body**	**Ant**	**Head**	**Ant.I**	**Ant.II**	**Ant.III**	**Ant.IV**	**Th.II**	**Th.III**
1243	464	300	50	135	82	197	178	129
**Abd.I**	**Abd.II**	**Abd.III**	**Abd.IV**	**Abd.V**	**Abd.VI**	**Man**	**Dens**	**Mucro**
111	100	129	407	89	100	264	161	68

#### Etymology.

From the name of the province “Songkhla” where this species was discovered.

#### Distribution.

Only known from the type locality.

#### Ecology.

Collected on guano in the dark zone of a cave developed in a karst covered with rainforest.

#### Discussion.

The new species is similar to *Cyphoderus javanus* Börner, 1906 and to *Cyphoderus sumatranus* Yoshii, 1987. The only detailed description of *Cyphoderus javanus* is that of *Cyphoderus borneensis* by Yoshii (1980, 1987), which was synonymized with *Cyphoderus javanus* by the same author in 1992. *Cyphoderus songkhlaensis* sp. n. differs from *Cyphoderus borneensis* as described by Yoshii in the following combination of characters: the posterior face of its ventral tube with chaetae L1 and L2 ciliated but M smooth (given however as ciliated in Yoshii 1980) (*versus* L1, L2, M all ciliated chaetae), anterior mac of ventral tube serrated *versus* smooth, spatulate *versus* blunt tenent hairs, no *versus* a few smooth basal chaetae on manubrium and claw with two inner teeth versus one inner tooth on claw.

*Cyphoderus songkhlaensis* sp. n. differs from *Cyphoderus sumatranus* by its ciliated clypeal chaetae (*versus* smooth in *Cyphoderus sumatranus*), the presence of one sublobal hair on outer maxillary lobe (*versus* none in *Cyphoderus sumatranus*) and the posterior face of its ventral tube with chaetae L1 and L2 ciliated but M smooth (*versus* L1, L2, M all ciliated chaetae). The new species is known from caves like *Cyphoderus sumatranus*, but *Cyphoderus javanus* has been reported from diverse habitats: termite nests, forest soil and caves.

### Cyphoderus khaochakanus sp. n.

http://zoobank.org/D18CFF8F-3003-4937-8DB3-C7CFF0C14E6D

http://species-id.net/wiki/Cyphoderus_khaochakanus

Fig. 8

#### Type locality.

Thailand: Sa Kaeo Province, Khao Chakan District, Tham Meud (Dark Cave) (13°39.541'N, 102°05.414'E), 73 m. above sea level, dark zone in cave, pitfall traps and Berlese extraction, S. Jantarit leg, 29 July 2012 (Sample #Sakaeo-SJ.001).

#### Type material.

Holotype, male adult (#PSUZC2011.SAK-001H) and 11 paratypes (1 male and 10 of unknown gender) mounted on slides. Holotype and 5 paratypes at PSU (5 slides, 5 subadults, collection #PSUZC2011.SAK-001P-005P) and 6 paratypes at MNHN (6 slides, 1 males, 5 subadults).

#### Description.

Habitus thick, not troglomorphic, body length about 1.3 mm excluding antennae and furca. Furca well developed, about 2.4 times shorter than body. Body color white. Eyes absent, no ocular patch. Dense cover of scales on head, body and furca (ventrally on manubrium, both sides on dens); scales present on Ant.I–II dorsally, absent on legs and ventral tube. Types of chaetae as in *Cyphoderus songkhlaensis* sp. n.

**Mouthparts.** Outer maxillary lobe with one basal chaeta, a simple palp and one sublobal hair. Maxilla with 3-toothed capitulum and a complex of 5–6 pad-shaped lamellae not analyzed in detail. Mandible head stocky, asymmetrical with 5 (left) and 4 (right) teeth. Labial palp with 5 papillae (A–E) and 13 guards, as in *Cyphoderus songkhlaensis* sp. n.; hypostomal chaetae (H, h1, h2) present; 4 proximal chaetae. Labial basis formula m,e,l1,l2, with all chaetae smooth or indistinctly serrated, and l2 reduced to a minute but thick mic. Labral formula 4/5, 5, 4 with all chaetae smooth; two chaetae of the mid-row stronger and longer than others (similar to *Cyphoderus songkhlaensis* sp. n.); dorso-distal limit of primary granules with a deep central incision; labral edge without structure; ventro-distally, two asymmetrical combs with many teeth variously developed and two central tubules.

**Antennae.** About 1.7 times the length of the head, segmentations I:II:III:IV as 1:3.6:2.5:4.8. Sens and sens-like chaetae present on all antennal segments, of 10 morphological types like in *Cyphoderus songkhlaensis* sp. n. (Fig. 3A); type-3 mes rather long, apparently smooth under microscopic examination. Scales present dorsally on Ant.I and II (like Fig. 3A11). Ant.I dorsally like Fig. 3B, with scales and ciliated mes (type-1), except 3 basal mic (type-9); ventrally like Fig. 3C, with various types of chaetae (types-1,2,3,5,6 and 9). Ant.II like Fig. 3D, E, both dorsally and ventrally with numerous slender sens and chaetae (types-1,3,4,5,6,7), with scales present dorso-basally; distally, 3–4 dorso-external swollen sens of type-7 and one ventro-external pseudopore. Ant.III like Fig. 3F, G, with various types of chaetae (1,3,4,5,6,7,9,10) not analyzed in detail; dorso-externally, AIIIO like Fig. 3H, typical of Entomobryoidea, with sens 1 to 5 and 8 easily recognized, 2 and 3 being swollen sens of type-7; ventro-externally, one subapical pseudopore. Ant.IV like Fig. 3I, J, devoid of apical bulb, with various types of chaetae (including all types of sens except type-10); subapical organ present dorso-externally as a short and thick rod.

**Dorsal chaetotaxy and pseudopores.** Patterns and types of chaetae similar to those of *Cyphoderus songkhlaensis* sp. n. (Fig. 4). Dorsal chaetotaxy and pseudopore patterns (per side) as follows: macrochaetae: 0/0,0/0,0,1,2 from head to Abd.IV (excluding the antenno-basal lines on head and the 7–8+7-8 lateral mac on Abd.IV) (Fig. 4B). Type-5 mes: not analyzed in detail. Trichobothria: 1/0,0/0,2,3,3,0,0 from head to Abd.VI. S-chaetae (sens of types S1, S2, S3 and S4): 0/2,1/1,2,3,4,3,0 from head to Abd.VI, arranged as in *Cyphoderus songkhlaensis* sp. n. Probably more S-chaetae on Abd.IV where type-5-like mes are often difficult to separate from S4. Pseudopores: 1–2/1,1/1,1,1,1+2,0,0 from head to Abd.VI.

**Ventral chaetotaxy of head.** 4+4 post-labial mes smooth or very finely serrated along linea ventralis, and one mic between G3 and H3 probably homologous with X (Fig. 2 in Chen and Christiansen 1993).

**Figure 8.**
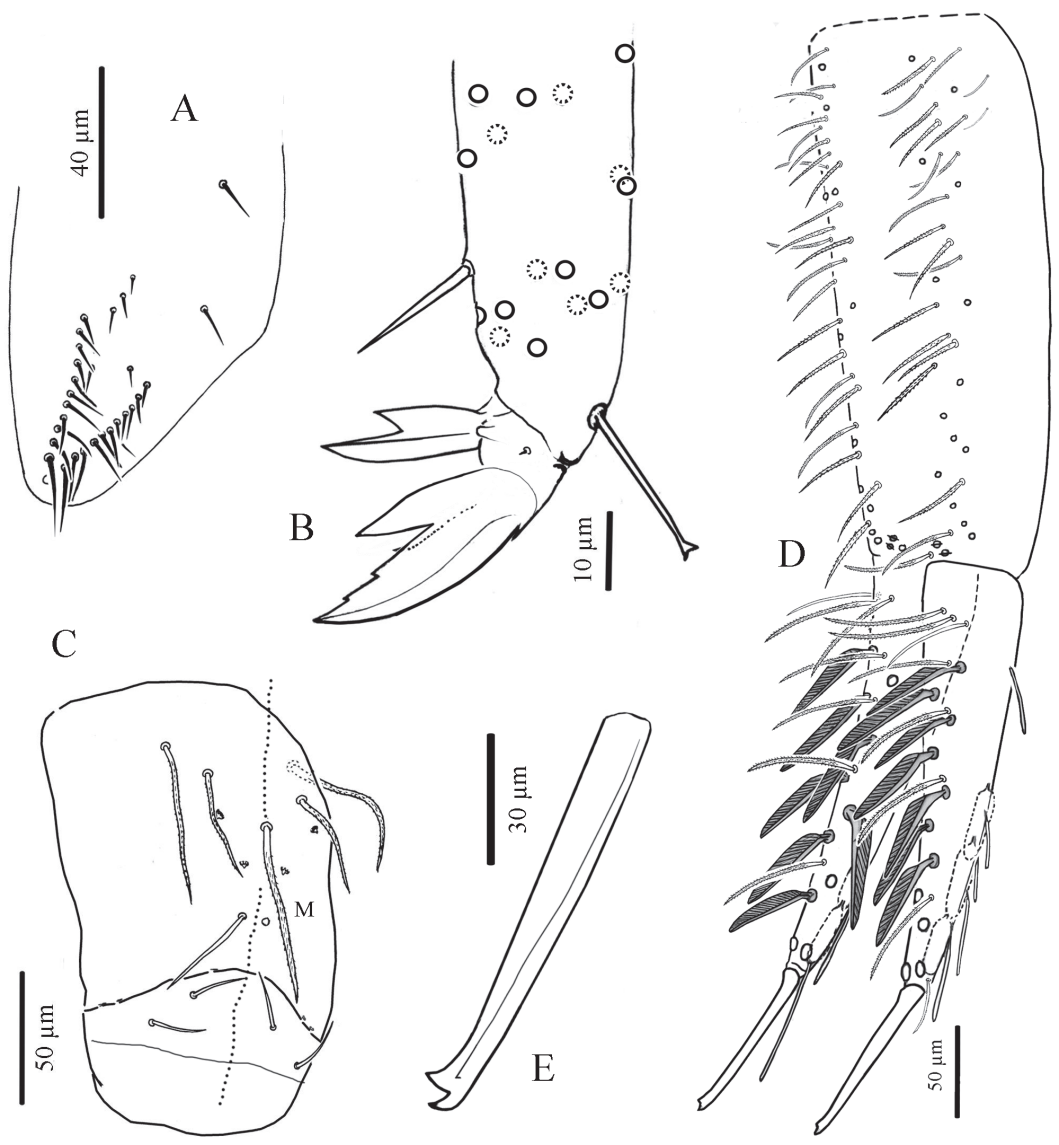
*Cyphoderus khaochakanus* sp. n. **A** trochanteral organ **B** claw and distal part of tibiotarsus III **C** posterior face of the ventral tube **D** furca; feathered chaetae in lateral view, only one of the two vanes attached to the rachis is visible **E** mucro.

**Legs.** Without scales. Trochanteral organ with 18 to 30 simple, straight, smooth chaetae arranged in V-form (Fig. 8A). Tibiotarsus chaetotaxy mostly composed of strong mes, with one thick smooth ventro-subapical chaeta on hind tibiotarsus. Each tibiotarsus with one tenent hair rather stout, apically spatulated, 3/4 to 4/5 as long as inner edge of claw; distal row of 9–10 serrated chaetae irregularly arranged on all tibiotarsi (Fig. 8B). Claw broad, not slender, without tunica; with 2 small teeth at 12% and 25% of inner edge from the tip of the claw, a small dorsal tooth basally and a pair of inner basal teeth of unequal size, the outer one much larger than the inner one (Fig. 8B). Unguiculus pointed and broad, more than a half as long as claw, lanceolate, with a strong outer tooth (Fig. 8B).

**Ventral tube.** Without scales. Anterior face with 2+2 long serrated chaetae (like Fig. 7F). Posterior chaetae arranged typically for the genus, with all 5 proximal chaetae (L1, L2 shorter than L1, M) ciliated, accompanied by 2+2 small peg-like microchaetae, and two long smooth distal chaetae; lateral flaps each with 2 short smooth mes (Fig. 8C).

**Furca.** Tenaculum with 4 teeth on each branch, and a strong, densely serrated, bent uneven chaeta anteriorly (like Fig. 7E). Furca with the same types of chaetae and scales as *Cyphoderus songkhlaensis* sp. n. (see Figs 7A, B). Manubrium slightly shorter or as long as mucrodens. Dens about 2.3 times as long as mucro. Dorsal side of manubrium (Fig. 8D) with 2+2 pseudopores distally, and about 32–35 mes (fallen in most cases) arranged in two longitudinal stripes, including rather flexible and strongly ciliated mes and a few lateral ones slightly stronger, more straight, feebly serrated (like Fig. 7A type-1), and baso-laterally 2+2 short serrated mes (like Fig. 7A type-3); ventral side covered with oval scales (like Fig. 7B5). Dens (Fig. 8D) elongate, dorsally with 2 rows of feathered scales (like Fig. 7B type-1), 6 external and 5 internal, and 4 ciliated mes (like Fig. 7A type-1) between the two rows; proximal outer part of dens with 3 chaetae, two ciliated (like Fig. 7A type-1) and the most external one smooth (like Fig. 7A type-2); proximal inner part of dens with 2 slightly serrated mes (like Fig. 7A type-1) close to the dens-manubrium articulation (like Fig. 7H); apical outer part of dens with a short serrated mes (like Fig. 7A type-3); long dorso-distal feathered scales fallen in our specimens. Dens ventrally with oval scales (like Fig. 7B types-4, 5), the two long distal ones fallen in our specimens. Mucro straight, elongate, bidentate apically, with an additional minute outer tooth almost at the level of the ante-apical normal tooth (Fig. 8E).

#### Measurements.

In µm (from type specimen #PSUZC2011.SAK-001H, male).

**Table:** 

**Body**	**Ant**	**Head**	**Ant.I**	**Ant.II**	**Ant.III**	**Ant.IV**	**Th.II**	**Th.III**
1316	545	328	46	164	114	221	221	121
**Abd.I**	**Abd.II**	**Abd.III**	**Abd.IV**	**Abd.V**	**Abd.VI**	**Man**	**Dens**	**Mucro**
93	86	107	528	96	64	328	243	96

#### Etymology.

From the locality “Khao Chakan” district, in SaKaeo province, where this species is found.

#### Distribution.

Only known from type locality.

#### Ecology.

Abundant on guano in the dark zone of a karstic cave.

## Discussion

*Cyphoderus khaochakanus* sp. n. differs from *Cyphoderus songkhlaensis* sp. n. by: 1) the claw with two inner unpaired teeth (*versus* one); 2) posterior face of ventral tube with all chaetae ciliated (L1, L2, M) (*versus* L1 and L2 ciliated, M smooth); and 3) manubrium slightly shorter than or subequal to mucrodens (*versus* manubrium longer than mucrodens). The number of teeth on the claw has been confirmed on 5 specimens of *Cyphoderus khaochakanus* sp. n. and 8 specimens of *Cyphoderus songkhlaensis* sp. n. Characters 2 and 3 are more difficult to observe, and their variability need to be assessed more firmly. In any case, these very slight differences are those usually reported in the literature between the species of the *albinus* group of *Cyphoderus*. Whether they indicate species-status would require re-examination of many species of the genus, especially for testing the variability of inner teeth on claw. We surmise that there are too few consistently different morphological characters in this group to further describe new species based only on morphology. We believe that molecular data will be helpful in providing additional information relevant to alpha taxonomy.

## Supplementary Material

XML Treatment for Cyphoderus

XML Treatment for Cyphoderus songkhlaensis

XML Treatment for Cyphoderus khaochakanus
